# Men and infertility in The Gambia: Limited biomedical knowledge and awareness discourage male involvement and exacerbate gender-based impacts of infertility

**DOI:** 10.1371/journal.pone.0260084

**Published:** 2021-11-29

**Authors:** Susan Dierickx, Kelvin Onyango Oruko, Ed Clarke, Sainey Ceesay, Allan Pacey, Julie Balen

**Affiliations:** 1 Research Center Gender, Diversity and Intersectionality, Vrije Universiteit Brussel, Brussel, Belgium; 2 School of Health and Related Research, The University of Sheffield, Sheffield, United Kingdom; 3 Faculty of Public Health Sciences, Kenya Medical Training College, Nairobi, Kenya; 4 Vaccines and Immunity Theme, Medical Research Council Unit The Gambia at the London School of Hygiene and Tropical Medicine, Fajara, The Gambia; 5 Safe Haven Foundation, Fajara, The Gambia; 6 Department of Oncology and Metabolism, The Medical School, The University of Sheffield, Sheffield, United Kingdom; Johns Hopkins University Bloomberg School of Public Health, UNITED STATES

## Abstract

**Introduction:**

Infertility in Sub-Saharan Africa constitutes an important social and public health problem. Yet, there is a paucity of research on the experiences of men living with infertility, especially in West Africa. This study explored men’s aetiological knowledge, views and experiences of infertility in the West Coast region of The Gambia, West Africa.

**Methodology:**

An explorative qualitative study was conducted among men living in the rural and urban communities of the West Coast region of The Gambia using in-depth interviews. Data collection and analysis were performed concurrently, and thematic data analysis was an iterative process carried out using NVivo 11 Analysis Software.

**Results:**

Gambian men had generally poor knowledge of infertility, allocating it to God, spiritual powers and bodily (biomedical) factors. While societal norms meant that infertility was generally attributed to women, some men allocated male-factor infertility to poor sperm quality and impotence. Infertility threatened participants’ sense of masculinity and resulted in psychosocial distress, including stigma, feelings of isolation, and low self-esteem.

**Conclusion:**

Normative gendered frameworks of infertility result in high levels of female responsibilisation in the Gambian context. Yet men diagnosed with infertility experience significant, often unrecognized, psychological and social distress. We therefore call for increased attention to male-factor infertility, and the promotion of male engagement with infertility-care and services, both of which are essential for successfully addressing infertility and it’s psychosocial consequences in The Gambia.

## Introduction

Scholarship on reproduction in sub-Saharan Africa has revealed that many communities have deep-rooted pronatal social norms, where the ultimate purpose of marriage is to bear children–emphasizing both a high desire, and significant social pressure to do so [1]. Yet, up to one in four couples in the region are confronted with infertility and the severe challenges it brings to daily life [2]. Indeed, infertility has a major impact on health and wellbeing, and it has been associated with several psychosocial problems in the African context and elsewhere [3–5]. For example studies conducted in Ghana, Nigeria, Rwanda and The Gambia have found that women who are unable to bear children are often stigmatised, isolated and disinherited, and may face physical and psychological distress and intimate partner violence [3, 4, 6–8].

Despite promises reiterated by the international community to improve the prevention and treatment of infertility, particularly among the most vulnerable, it remains a neglected component of global reproductive health research, policy and practice [9, 10]. Access to appropriate infertility care and services is severely limited across sub-Saharan Africa, despite a steep rise in use of assisted reproductive technologies elsewhere [11]. While cost is a major barrier in resource-limited health systems, some approaches–including early diagnosis and treatment of reproductive tract infections–are relatively simple and cost-effective [12]. This is particularly pertinent in sub-Saharan Africa, where untreated infections and subsequent pelvic inflammatory disease, are thought to be the main causes of infertility [3, 13, 14].

Historically, women have been held responsible for a couple’s infertility, with a majority of public health and social science research focussing on the experiences and care seeking behaviours of women (compared to those of men). More recently however, there has been a growing awareness of male-factor infertility, as well as of the importance of male engagement in infertility services [12, 15, 16]; in The Gambia this is partly due to ongoing reproductive activism [13]. Of the emerging literature that has addressed infertility among men, relatively few studies have focused on men in West Africa (with some exception), and no studies conducted to date in The Gambia [7, 17]. This invisibility of African men is problematic because it reinforces the idea that women are responsible (and to be blamed) for infertility. It also contributes to the ongoing concealment of reproductive health care needs of men who face (male- and/or female-factor) infertility within their marriages, and to the lack of appropriate male-oriented responses [4, 16].

Growing clinical attention paid to infertility in The Gambia, and considerable gender-based health inequities, call for increased male involvement in infertility care and services [3, 13]. Here, we present findings related to men’s aetiological knowledge, views and experiences of infertility, and the impact it has on their lives, in the West Coast region of The Gambia, West Africa. This study was conducted as part of a larger anthropological and health systems research programme designed to understand the lived experiences and access to appropriate care among people with infertility in Senegal (Casamance) and The Gambia (West Coast region) [3, 13, 18–20].

## Methods

### Study site and population

This qualitative study was conducted in both rural and urban communities within Brikama and Kanifeng Local Government Areas, in The Gambia. Our earlier work showed that The Gambia is a strongly pronatalist society, with the desire for childbearing informed by social, economic and emotional factors [18]. There is scant information on the prevalence of infertility in The Gambia, in particular male-factor infertility. One study, conducted two decades ago, reported an estimated 3% primary infertility rate among women, and 9% of women experiencing subfertility (measured as three years without bearing children despite being married, no contraceptive use or breastfeeding) [21].

### Sampling, recruitment and sample characteristics

Standard epidemiological and demographical conceptualisations of infertility are not always meaningful for people living in low- and middle-income countries [18, 22, 23]. Anthropologists have shown how conceptualisations about what is natural, normal or expected in terms of fertility varies historically and geographically. Previous research in The Gambia illustrated that infertility is often allocated to women (i.e. presumed female-factor), regardless of whether any formal diagnosis is conducted [3, 18]. Moreover, in general, men are reluctant to attend clinics when faced with infertility in their marriage [3]. Therefore, men were included in this study if they were in a marriage wherein fertility problems have occurred, regardless of whether they themselves were formally diagnosed or not, and regardless of whether it was male- or female-factor infertility, or unexplained.

Since infertility is a highly stigmatised condition within this setting [18], participants were identified through snowball sampling, whereby respondents identified other potential respondents. The sample included 13 men from different ethnic groups, locations, religious backgrounds and ages (Table 1). Eleven participants were in monogamous marriages, one man had two wives and another man had four wives. While all participants had previous or ongoing fertility problems within their marriage(s), five were childless (primary infertility), and eight had at least one child but faced difficulties upon subsequent attempts (secondary infertility). The majority of the respondents were between 21 and 50 years. In terms of education, the highest education respondents received was tertiary level, while four respondents had no formal education. Most respondents were Muslim which is the common religion in the country.

**Table 1:** Study participants’ key characteristics, including age category, ethnic group, religion, type of marriage and number of biological children.

Category		Participants (N = 13)
		n
Age group		
	Adult (21–50 years)	9
	Elder (+ 51 years)	4
Ethnic group		
	Mandinka	6
	Fula	3
	Wolof	1
	Aku	1
	Karoninka	1
	Manjago	1
Religion		
	Muslim	10
	Christian	3
Type of marriage		
	Monogamous	11
	Polygynous	2
Number of biological children		
	*Men in monogamous marriage*	
	0	5
	1	4
	2	1
	3	1
	*Men in polygynous marriage*	
	5	1
	8	1

### In-depth interviews

Semi-structured interviews were conducted in English or in a local language by KOO or SD with the support of a trained translator, and were audio-recorded with consent. Discussion centred around the following topics: (i) societal views of infertility; (ii) participants’ perceptions and knowledge of infertility including male- and female-factor infertility; (iii) their care seeking behaviours (reported elsewhere); (iv) impacts of infertility on their daily lives; and (v) their coping strategies. An interview guide was used for the interviews (S1 File). Where required, probing followed the responses given. The interview guide was adapted according to emerging results, the reproductive history of the participants as well as their indications of comfort talking about certain topics. This latter aspect was mainly deducted from non-verbal communication by the respondents such as their body language, gestures, interests and prolonged silences.

Prior to data collection, the translator received training in the topic of interest, research facilitation, interview skills and confidentiality protection. During this training phase, the translator was supplied with the interview guide in order to discuss appropriate terminology, phrasing and translation of potential questions. The interviews were conducted in the place and time the participants found convenient, this was in practice the privacy of their homes or workplaces. The interviewers and interviewees ensured that nobody could overhear the conversation with the exception of three cases whereby the husband and wife explicitly requested to be interviewed together. Non-English interviews were transcribed in the language used during the interview and then translated into English. The transcripts were then checked and confirmed by an experienced translator from the wider project by listening to the audio recording while reading the English language transcripts.

### Reflection on positionality

KOO is a black African male, who is professionally trained in public health, with eight years of experience conducting research on highly sensitive and taboo topics in sub-Saharan Africa [24, 25]. Combining his positions of insider (African male, MRC Gambia affiliation) and outsider (non-Gambian, UK university affiliation) facilitated a more rapid acceptance by the participants. SD is a white woman who is trained as a sociologist and anthropologist. She has been conducting research in rural and urban communities in The Gambia for almost a decade on a variety of topics, including infertility. Due to this experience, she is acquainted with culturally appropriate behaviours and has an extensive network. SD was familiar with the interviewed men before the research, and they approached her privately to request an interview with them about their experiences. The translator was a Gambian man trained in research ethics and interview techniques living in the urban area but not previously familiar with the participants. His presence as a translator was often necessary and did not appear to influence the results.

### Data analysis

Qualitative data collection and analysis were performed concurrently, and thematic data analysis was an iterative process. Analysis consisted of repeatedly reading each transcript, identifying statements and quotes, and then labelling and connecting these to particular themes. Through this process, data were subjected to theoretical perspectives in order to embed the findings within existing literature. The systematization and analysis of all qualitative data was carried out using NVivo 11 Analysis Software.

### Ethical approval

This study was approved by The Gambia Government/MRC Joint Ethics Committee (SCC1562), and by the ethical commission of Vrije Universiteit Brussel (ECHW_096), Belgium, and the Research Ethics Committee of The University of Sheffield School of Health and Related Research (ScHARR) (018955), United Kingdom. The researchers followed the Code of Ethics of the American Anthropological Association. The researchers explained to the respondents that their participation in the study was fully voluntary. Measures were taken to ensure confidentially and privacy. Access to recordings and transcript was limited to the research team. Written informed consent was obtained from all respondents prior to each interview. In order to ensure anonymity only the age category and an indication of whether the participant is confronted with primary or secondary infertility in their marriage is presented.

## Results

### Aetiological knowledge of infertility

#### Non-bodily causes of infertility

Most respondents stated that children are from God, and childlessness is God’s will. One of the respondents, who was of Christian faith, also indicated that childlessness was seen as a punishment of sins:

*‘You know children are a blessing from God and when you are not able to get them*, *then people may say that you are in sin or you are not walking in the right path*.*’* [Adult man, secondary infertility]

Additionally, it was thought that both men and women could be affected by spiritual causes of infertility, including black magic used by Marabouts (i.e., indigenous healers who heal but can also cause harm), *buwaa* (witchcraft), and evil spirits. In general, the perception is that these spiritual causes are more likely to affect women, compared to men, as they are perceived to have ‘weaker’ bodies and minds enabling spiritual attacks:

*‘I believe this lack of children is something caused by tradition*, *like someone is trying to play with our marriage*. *It is some spirit*. *Even if you don’t believe it*, *I know there are these spiritual things that can cause infertility*.*’* [Elderly man, primary infertility]

#### Bodily female-factor infertility

Overall, men had limited knowledge about potential causes of infertility among women. Some men mentioned *seketoo* (i.e., a folk illness resembling urinary tract infections) and vaginal infections. Others reported that some women could have a *‘wrong female body or shape’* and problems with the womb, which can be *‘tight’* or *‘weak’*:

*‘… so*, *that warranted her to go the clinic again*, *to the midwives to do the examination and they* [the midwives] *told her the placenta*, *the womb is weak*. *So*, *it cannot hold the embryo*, *that anytime it starts developing then it falls off*.*’* [Adult man, primary infertility]

Some men attributed infertility problems to poor timing of conceiving and the lack of ovulation. Other causes of infertility mentioned were related to past behaviour of women, including the lack of female circumcision causing infertility problems, the late age of marriage and the use of contraception ‘*blocking*’ or otherwise affecting the womb. Two lifestyle factors affecting female infertility were smoking which was said to *‘break eggs’*, and engaging in sports.

#### Bodily male-factor infertility

In relation to men’s knowledge about male-factor infertility, impotence and sperm quality were commonly mentioned. However, several respondents confused these two factors and assumed they were equivalent issues. Generally, men with a higher educational status had more knowledge about hormones and lifestyle factors affecting male-factor fertility, including diet and smoking. Details of more formal lab-based diagnoses, among the five—all higher educated—respondents who had sought biomedical care, were sperm problems, an infection, or the diagnosis that the cause of their infertility remained unknown. Three of them associated their infertility with conditions they had suffered previously or currently, such as haemorrhoids, physical weakness and diabetes:

*‘She has no problem*. *So*, *I believe the problem is on me*. *And that is my worry*, *the sperm problem*. *And I can feel something on my testicles*, *one side is bigger than the other*. *Sometimes I can feel some little pain*. *I wonder why this*?*’* [Adult man, secondary infertility]

*‘It was a surprise to me actually that I was diagnosed with a low sperm count*, *because I am always full and strong and [with much] energy*. *Just recently I got diagnosed with diabetics [diabetes] and I am on diabetic treatment*. *It could be that it has some impact on me*.*’* [Adult man, secondary infertility]

#### Bodily “interpersonal” infertility

In addition to bodily causes allocated to either men or women, several participants reported reasons related to the specific combination of partners, including the incompatibility of blood among husband and wife, genetic factors and unspecified diseases or pathology affecting both partners:

*‘Some perceive that there is* [blood] *incompatibility and they can advise you to get a second wife’* [Elderly man, secondary infertility]

### Gendered interpretations and experiences of infertility

Apart from the three men who had been formally diagnosed and specific causes were identified, respondents often allocated infertility to their wives, and to women in general. This reflects a socio-cultural pattern whereby infertility is commonly attributed to women by the family and society. Yet, there are some exceptions when family and society do not allocate infertility solely to women, for example when a newly married young woman with an older husband is not able to conceive; or when a man marries multiple wives, and all are not able to conceive with him. However, even then, with time the blame may shift to the women if they remain in the marriage without giving birth. While respondents agreed that women are blamed for infertility and consequently suffer more, they also perceived that women with infertility received more social support in contrast to men with infertility who, despite usually not being held responsible for ‘causing’ or having to address infertility, nevertheless faced consequences including: (i) stigmatization within their communities; (ii) challenges within their marriages and family dynamics; and (iii) a negative impact on the mental health.

#### Stigmatization within the community

Despite infertility was commonly attributed to their wives, all respondents mentioned that their masculinity was questioned by society at some point and some men with infertility reported being stigmatised:

*‘It has disturbed my mind because in our society when one is in such a condition* [childless] *people talk* [gossip] *a lot about it*. *I am feeling it and it has disturbed my mind’* [Adult man, secondary infertility]

Male experiences of stigmatisation included being scorned or mocked in the community through the use of phrases such as ‘*you are not functioning’*, ‘*you are a weak man’*, ‘*you are useless*’,‘ *you do not have sex properly’* and by calling men with infertility by the name of their wife which is perceived to be very derogatory in this setting:

*‘They start to call you*, *‘oo wonna gorko’ (you are not a man)*. *They start to call you with your wife’s name*.*’* [Adult man, primary infertility]

*‘They will say this man is not a real man*.*’* [Adult man, secondary infertility]

#### Impact on marriage and family dynamics

All respondents noted that infertility leads to destabilization of families, resulting in divorce, polygyny, and extramarital affairs. These were said to occur especially when it is believed that the cause of infertility is ‘*blood incompatibility’*, or when it is suspected to be female-factor infertility which, as outlined above, is often suspected. There is considerable pressure on men to engage in polygyny (in case of Muslims) or to divorce (in case of Christians) because of: (i) broader societal beliefs whereby it is perceived those men who do not want to re-marry when a first marriage is childless are infertile themselves; and (ii) pressure by family members or friends.

In this study sample, most respondents were reluctant to engage in polygyny or divorce because: (i) they suspected or were aware that they (themselves) had male-factor infertility; and (ii) they feared tensions within their (new) marriage. While extramarital affairs are initiated by some men who face infertility, or by women when they suspect that the problem lies with their husband, this is not permitted within the community or religion rather encouraging men to instead engage in polygyny. Nevertheless, extramarital affairs were said to be a common silent behaviour.

#### Impact on mental health

When participants were asked about their feelings related to infertility, their difficult and painful emotions were evident. Men were said to have little choice but to suffer alone and in silence. All men felt sad, stressed, emotionally drained, ashamed, unconfident among peers and having poor self-esteem. In addition, some respondents expressed jealousy and feeling excluded from social and religious events, where people talk about their children. These feelings originated from infertility itself, as well as from everyday challenges that come with it:

*‘… my feelings of not having children*! *Sometimes I feel bad*, *because in our family we are seven children*, *mostly boys and am the second eldest of the family*. *Three of my younger brothers are married and they have children*. *Sometimes it keeps me shy*: *like they meet their children and me I don’t have children*, *so sometimes it feels too bad*.*’* [Adult man, primary infertility]

### Coping strategies

Men who are diagnosed as infertile do not always share this information with their wife/wives or family members, out of fear of what might happen to their marriages and how extended family members but also wider society will perceive them.

*This is the first time I have an opportunity to talk about this thing* [infertility], *we rarely talk about it*.*’* [Adult man, primary infertility]

Some respondents also avoided unnecessary contact with family members and the broader community. This is not only a way to cope with stigmatisation and mental health problems but also a strategy to cope with the difficulties caused by infertility within their marriage, for example some respondents lived apart from their extended family who would otherwise pressure them to engage in polygyny or divorce. Participants further noted that marriages do not always deteriorate when infertility occurs. Several respondents reported that they supported their wives and loved them more deeply because of infertility, or that they felt supported by their wife despite having been diagnosed with infertility:

*‘We love each other when we were both healthy*, *and then after marriage we realized that this infertility problems are developing*. *I see this as a temptation and I can overcome it*. *I can go through it*, *she also understands that*. *Even if I still remain like that* [i.e. he is diagnosed with low sperm count] *she will stay in love*.*’* [Adult man, secondary infertility]

Men will also try to overcome the childlessness by looking for spiritual and biomedical health care as well as by fosterage. Fostering is a common practice in The Gambia happening commonly outside a legal framework, two respondents had fostered children of close relatives, and they raised them as their own.

## Discussion

This is the first study of its kind in The Gambia, and to our knowledge across much of West Africa, providing a detailed, albeit introductory, account of male perceptions and experiences of infertility. Men had generally poor biomedical knowledge of infertility, which they thought was caused by different spiritual or bodily factors such as ‘blood incompatibility’. While infertility was said to be broadly attributed to women, male-factor infertility was culturally constructed as a man’s inability to sexually function and satisfy a woman, as well as his inability to impregnate her. We found a general belief that poor sperm quality and impotence are causes of male-factor infertility, which is in line with claims that in some cultures, for example, among the Sara of Chad and Yoruba of Nigeria, a man is considered to be infertile if he is impotent [26, 27]. In our study, infertility threatened participants’ sense of masculinity and resulted in psychosocial distress, including stigma and low self-esteem. While support for men with infertility is limited in The Gambia, and marriage-breakdown was said to be a common outcome, men employed different coping strategies and mechanisms for self-management, including moving to another community, keeping it a secret, limiting social engagements, and strengthening their marital bond.

A number of different spiritual, social and bodily causes of infertility were mentioned, many of which have been reported elsewhere [28]. There is a general lack of biomedical knowledge of infertility among men, which might impede prevention and timely care seeking of both members of the couple, and leads to the blame and responsibility for infertility being placed primarily on the female [17]. Although it was acknowledged that it is normative that infertility is regarded as a woman’s problem within Gambian communities, participants mentioned causes of infertility that lie within the male partner (i.e., male-factor infertility), or female partner (i.e., female-factor infertility), as well as both (i.e., different forms of relational incompatibility) or neither (i.e., unexplained infertility). This differs from work conducted in The Gambia 20 years ago which reported that infertility was regarded as only female-factor [29]. This development may illustrate changes in health literacy, fertility care services and perhaps gender norms, in The Gambia over that time. A similar development was noticed in rural northern Malawi due to the spread of biomedical knowledge on male reproductive health [30].

While our previous research in The Gambia illustrated that women with infertility experience severe stigmatization within their community [18], studies conducted in Zimbabwe, Malawi and South Africa that included male participants found that men are also at risk of infertility-related stigma [17, 30, 31]. Moreover, both males and females across all societies experience deep and intense trauma and loss, including emotions related to grief, shame and sadness, when faced with infertility [4]. We found that men with infertility reported suffering from social anxiety and low self-esteem, as well as feeling deprived of ‘full manhood’. Further research could delve into the impact of diagnosis and treatment of male-factor infertility on men’s experiences on their emotional well-being and their relationships within their marriages and communities [30]. Feelings of sexual inadequacy can amplify such negative emotions, especially among men who also struggle with impotence. Provision of male counselling services, considering both spiritual and biomedical concepts of infertility, is therefore critical, both in order to support men with infertility, and to provide them with the tools to adequately support their wife/wives–which many participants expressed a desire to do, but felt inadequately equipped for. We argue that the inclusion of men in infertility programmes may help address issues of family breakdown and interpersonal violence, which we came across anecdotally.

Creating awareness and providing accurate information about infertility, its causes, and prevention and treatment options, is important in addressing the issue. This might include encouraging infertility to be viewed as a relational “couples” issue, requiring a shared approach to prevention and treatment for both women and men. Exactly how to do this remains a topic of inquiry, given the sensitivities outlined above. We propose gender-based health education on (fertility and) infertility, including biomedical causes and risk factors, coupled with further investigation on how awareness can be generated within different contexts and how different types of knowledge can influence care seeking behaviour.

Though our data is limited to a small qualitative sample in the most urbanized region of The Gambia, it is the first of its kind focussing on the voices of men with infertility. The sample is not representative of the entire population and therefore the results cannot be generalized across the country, yet they shed light on an under-researched area of male perspectives and experiences of infertility, especially in sub-Saharan Africa. While we did face some difficulties in recruiting men (as have other studies of this nature), those who participated offered deep and candid accounts [32, 33]. The trust and comfort built helped them open up and share their stories/experiences on such a highly sensitive topic, rarely spoken about within African societies.

## Conclusion

This study has provided an important, though preliminary, understanding of men’s perceptions and experiences of infertility in The Gambia. It sheds new light on the role of masculinity in reproductive health within sub-Saharan Africa, where normative gendered frameworks of reproduction result in continued female blaming for infertility, though perhaps less so than in previous infertility studies conducted 20 years ago. We call for increased focus on male factor infertility, and increased male engagement in infertility-care and provision of services, both of which are essential for successfully addressing this hitherto neglected reproductive health issue in The Gambia and beyond.

## Supporting information

S1 File Question guide. (PDF)
