# Supplementary material for: Men and infertility in The Gambia: Limited biomedical knowledge and awareness discourage male involvement and exacerbate gender-based impacts of infertility
Source: PLoS One. 2021 Nov 29;16(11):e0260084. doi: 10.1371/journal.pone.0260084 (PMC8629172; doi:10.1371/journal.pone.0260084)
Supplement: S1 File — (PDF) [file pone.0260084.s001.pdf]

Introduction

I am ____________________working for "Infertility and relationships in The Gambia" study. I welcome you in this interview. I will be the moderator for this interview and Mr. ______________________is my colleague whom we work together with. He will be taking notes and recording audio during this session.

Feel free to make negative or positive comments about any of the things we'll be talking about today. This is a free-flowing session and there are no wrong answers. What you say will not bother me, so please feel free to speak your mind.

Purpose

This interview aims to explore your experience as a man who is currently childless. You have been invited for this interview because your input is essential in understanding how men manage to cope with childlessness. We will initiate our discussion with general questions about your perception of infertility. We will then talk about the care seeking behavior, motivation and barriers, and coping strategies.

This interview is expected to take ¾ – 1½ hours.

Ground Rules

We will be recording this interview to ensure we do not miss any of the responses, and to write accurate report afterwards. We will also be taking notes throughout the interview for backup. All information recorded will be kept confidential and you will not be identified by name. This information will be used for the sole purpose of this research. You may choose not to respond at any time.

ICE BREAKER: What is your role as a man (husband) in marriage?

Themes

THEME I: **Perception about infertility**

- Do people talk about infertility openly
    - [if yes, what do they say]
- How would you describe infertility?
- What names are used to refer to infertility in this community
    - What of men [or women/couples] who are infertile

THEME II: **Knowledge of Infertility** [linked to theme I]

- Causes of infertility
    - In your opinion what do think might have resulted to your infertility [a bit sensitive]
        - What did healthcare providers tell you as the probable cause [might want to ask after getting to care seeking behaviour]
- When did you realize that you have an infertility problem?
    - Is it confirmed [by who?]
    - Have you shared with your wife about it?
        - What of the family [extended]
        - Other people like friends

THEME III: Care seeking behaviour

- Where do you seek for care?
    - Do you seek for care in solo or with your wife together?
    - Traditional care
        - What happens during the care
    - Biomedical care
        - What services/treatment did [do] you receive
            - Counselling
    - Others: are there other places that other childless men seek for care?
        - Spiritual leaders
        - Secret places
- When did you start seeking for care?
    - How often do you seek for care [how many times have you sought for care]
        - Who seeks care more often between you and your wife
- What motivates you to seek for care?
    - [also: who motivated you? – wife, friends, ..]
- What challenges [barriers] do you face when seeking for care?
    - Cost
        - For the traditional care and biomedical care
    - Fear [feeling ashamed]
- Are there any groups that men with infertility problems join?

- [Like Kanyaleng for women]
- What services/support do you get from the group?

- **THEME IV: Impacts of infertility**

  Social and psychological impact

  - How do you feel for this condition of childlessness?
    - Do you feel stigmatized
  - How does your wife feel about it?
    - How has childlessness affected your relationship with your wife
  - How does the family [extended] feel about it?
    - Your [man's] family
    - Wife's family
  - How does the community treat people [men/couples] who are childless?
    - Your interaction with fellow men
    - Your interaction with women
    - Are there community events that you are not allowed to attend?

  Economic impact

  - How has it affected you financially?
    - Cost of treatment
    - Work [work time converted to search for care]

  Legal impact

  - Are there any legal issues that you face due to childlessness?
    - Inheritance of property [for you or wife]

THEME V: Coping strategies

- What do you do to cope up with childlessness?
- Adoption of a child
- Marrying another wife
  - Having a relation outside marriage [*sensitive – ask about other men]*

THEME VI: Desire for children [linked to the icebreaker]

- Before you knew about your infertility problem how many children had you desire for
  - Any gender preference
  - **Has the desire changed now?**
    - **If the problem were solved how many children would you**

have

- **Why do men [you] desire to have children**

END OF THE INTERVIEW

CLOSING COMMENTS

I am very thankful for agreeing to participate in this important interview and valuable time that you spared. Your comments are very important to this study to better understand the care seeking behavior and mitigation strategies used by men. Please feel free to ask us any questions you may have.
